# Impact of mid-season sulphur deficiency on wheat nitrogen metabolism and biosynthesis of grain protein

**DOI:** 10.1038/s41598-018-20935-8

**Published:** 2018-02-06

**Authors:** Zitong Yu, Angela Juhasz, Shahidul Islam, Dean Diepeveen, Jingjuan Zhang, Penghao Wang, Wujun Ma

**Affiliations:** 1 State Agricultural Biotechnology Centre, School of Veterinary and Life Science, Murdoch University, Perth, WA 6150 Australia; 2 Western Australian Department of Agriculture & Food, 3 Baron-Hay Ct, South Perth, WA 6151 Australia; 3 Australia-China Joint Centre for Wheat Improvement, Murdoch University, Perth, WA 6150 Australia

## Abstract

Wheat (*Triticum aestivum*) quality is mainly determined by grain storage protein compositions. Sulphur availability is essential for the biosynthesis of the main wheat storage proteins. In this study, the impact of different sulphur fertilizer regimes on a range of agronomically important traits and associated gene networks was studied. High-performance liquid chromatography was used to analyse the protein compositions of grains grown under four different sulphur treatments. Results revealed that sulphur supplementation had a significant effect on grain yield, harvest index, and storage protein compositions. Consequently, two comparative sulphur fertilizer treatments (0 and 30 kg ha^−1^ sulphur, with 50 kg ha^−1^ nitrogen) at seven days post-anthesis were selected for a transcriptomics analysis to screen for differentially expressed genes (DEGs) involved in the regulation of sulphur metabolic pathways. The International Wheat Genome Sequencing Consortium chromosome survey sequence was used as reference. Higher sulphur supply led to one up-regulated DEG and sixty-three down-regulated DEGs. Gene ontology enrichment showed that four down-regulated DEGs were significantly enriched in nitrogen metabolic pathway related annotation, three of which were annotated as glutamine synthetase. The Kyoto Encyclopedia of Genes and Genomes pathway enrichment identified three significantly enriched pathways involved in nitrogen and amino acid metabolism.

## Introduction

Wheat (*Triticum aestivum*) grain quality is mainly determined by grain storage protein composition. Increasing protein content and optimization of protein composition are two common approaches in targeting wheat quality improvement. Both sulphur and nitrogen are essential macronutrients and building blocks of protein biosynthesis. Many reports attest to the capability of nitrogen fertilizer to increase protein content while sulphur fertilizer affects protein composition^1–3^. As we have learned in recent decades, the availability of sulphate in the soil is becoming a limiting factor for plant growth. Plants assimilate sulphur using sulphate transporters capable of taking up inorganic sulphur from the soil and translocating it to other organs to be incorporated into multiple organic compounds. This is achieved through a concerted regulation of sulphur metabolic pathways during the plant’s whole life cycle^4,5^. Without an adequate supply of sulphur, wheat is not able to reach its full yield potential and make efficient use of nitrogen for protein biosynthesis. Improvement of nitrogen use efficiency (NUE) has been a major aim of recent agricultural research, since nitrogen fertilizer has become the largest input cost and its price continues to increase, driven by demand and production costs. On the other hand, nitrogen runoff from agricultural lands threatens the environment, affecting the quality of air, water and soil. Because of the interaction between environmental effects and genetic factors, the enhancement of NUE is complex^6^. The average ratio of nitrogen and sulphur in proteins is twelve to one and plants usually accumulate more than 80% of reduced nitrogen and sulphur compounds for protein biosynthesis at a rather constant ratio. Therefore, the accumulation of glutamine, asparagine and arginine during sulphur starvation may reflect the removal of surplus nitrogen. A reduction nitrate reductase (NR) activity during sulphur starvation seems to be a consequence of transcriptional down-regulation^7,8^.

Nitrogen assimilation begins with nitrate uptake by the roots and transport to the shoots by nitrate transporters. During this process, NR reduces nitrate to nitrite in the cytoplasm, and subsequently nitrite is reduced to ammonium by nitrite reductase (NiR) in the plastids. In addition, a certain amount of ammonium is directly transported by ammonium transporters (AMTs). Ammonium is further assimilated into glutamine (Gln) and glutamate (Glu) by glutamine synthetase (GS) and glutamate dehydrogenase (GDH) through the GS/GOGAT cycle^9–11^. Glutamine can be converted into asparagine (Asn) and glutamate (Glu) by asparagine synthetase (AS) and glutamate synthase (NADH-GOGAT). Besides GS, AS is another critical enzyme in the primary nitrogen metabolic pathway^10,12^. Overexpression of NADH-GOGAT results in a maximum increase in grain weight by as much as 80%. Unlike ferredoxin (Fd)-GOGAT, involved in photorespiration, NADH-GOGAT gene is active in developing organs such as unexpanded non-green leaves and developing grains, and involved in nitrogen remobilization from both primary and secondary sources. Thereupon, Gln, Glu and Asn can enter the biosynthetic pathways of other amino acids as substrates for various aminotransferases, depending on the plant’s developmental needs^13,14^. Glutamine synthetase (GS) is the key enzyme for nitrogen metabolism, catalysing the assimilation of all inorganic nitrogen for its incorporation into organic compounds such as proteins and nucleic acids. This reaction is coupled with the formation of glutamate by GOGAT as part of the GS/GOGAT cycle^15^. GS exists in multiple enzyme forms, with the chloroplastic isozyme encoded by one gene and the cytosolic form encoded by three to five genes, depending on the plant species. The different isoforms are regulated during plant growth and assume different roles in the glutamine metabolic pathway. In wheat, the three major *GS* genes are located on different chromosomes and play different roles during plant development. *GSr* is located on chromosome 4 A and has ability to regulate nitrogen remobilization and total GS activity in wheat. The expression of the *GSr* gene increases during the later stages of leaf development, being one of the key genes involved in nitrogen remobilization in senescent leaves. The *GS1* gene is located on chromosome 6 A and its cytosol-located product has multiple metabolic functions such as assimilation of ammonia into glutamine for transport and distribution throughout the plant. The *GS2* gene is located on chromosome 5D and its product plays a vital role during the vegetative stage. GS2 is the predominant isozyme in leaf mesophyll cells and assimilates ammonia originating from nitrate reduction and photorespiration^16–18^.

Previous study reported that sulphur metabolic processes start with sulphate uptake by the corresponding transporter^19^. Subsequently, sulphate is activated by covalent binding to ATP via an ATP-sulphurylase-catalysed (ATPS) reaction to form adenosine 5′-phosphosulfate (APS). APS is reduced to sulphite by APS reductase (APR) and then sulphite is reduced to sulphide by sulphate reductase (SiR). The sulphide is then transferred to activated serine by O-acetylserine (thiol) lyase (OASTL) to form cysteine^20^. It is important to note that serine acetyltransferase (SAT) is the rate-limiting enzyme in cysteine biosynthesis^21^. Serine is converted into O-acetylserine (OAS) by the catalytic activity of SAT while bound to OASTL in a multi-enzyme mixture known as OASTL-SAT mixture. OASTL, on the other hand, only becomes active in cysteine biosynthesis once released from the complex^22,23^.

Cysteine is the end product of sulphur metabolism and mainly responsible for the formation of disulphide bonds, which play a major role in protein aggregation, thus providing the main mechanism behind viscoelasticity of the dough matrix^24,25^. Different storage protein subunits contain different numbers of cysteine residues capable of forming inter- or intra-molecular disulphide bonds. Apart from this, cysteine is a precursor of several essential sulphur-containing compounds, such as methionine and compounds involved in resistance to environment stresses, such as glutathione (GSH), S-adenosylmethionine (SAM), S-methylmethionine (SMM), and glucosinolates^26^.

Glutathione (GSH) is the main transport and storage form of reduced sulphur in plants. It has the ability to regulate plant growth by modulating processes such as mitosis, cell elongation, resistance to environmental stresses, maintaining the redox homeostasis and detoxification^27^. Previous studies have reported that GSH was particularly important in tolerance and adaptation to certain abiotic stresses. Changes in the GSH pool provide an indication of the redox state of the cell, which might influence the expression of important genes involved in responses to environmental stresses. Specifically, increases of the GSH pool have been observed in response to biotic and abiotic stresses, including pathogen attack or heavy metals stress. However, plants with a diminished GSH pool were more sensitive to a range of environmental stresses, such as heavy metal and oxidative stresses^28–30^. In contrast to GSH, glucosinolates have been reported to be specifically involved in resistance to biotic stresses, such as in pathogen defence mechanisms in which also the largest transcription factor family, the MYB superfamily, is involved. Specifically, MYB28, MYB29 and MYB76 are capable of regulating aliphatic glucosinolates, which are derived from methionine. However, MYB51, MYB34 and MYB122 have the ability to control indolic glucosinolates, which are synthesized from tryptophan. The MYB transcription factors themselves are regulated by jasmonate^31–33^. SAM is a source of reactive 5′-deoxyadenosyl radicals used by numerous enzymes, and the aminoisopropyl group for the synthesis of polyamines and biotin. SAM is also a donor of methyl groups involved in the biosynthesis of amino acids, nucleic acids, and a precursor of ethylene, nicotianamine, and phytosiderophores. SMM in cereals has been reported to be probably used in long-distance transport of reduced sulphur^34,35^.

A sufficient supply of sulphur is a key factor due to the complex interactions of sulphur and nitrogen in protein biosynthesis and the vital roles of sulphur metabolites in wheat plant growth and grain development. Therefore, to understand the effects of sulphur starvation on nitrogen metabolic pathways and protein biosynthesis as well as the consequences on relevant agronomic traits and protein quality, we conducted a transcriptomics analysis by comparing plant responses to high and low sulphur treatments. Several transcripts responsive to the treatments were identified and their functions in nitrogen metabolic pathways elucidated using gene ontology annotation and KEGG pathway enrichment analysis.

## Results

### Agronomic traits and protein parameters

**Figure 1:**
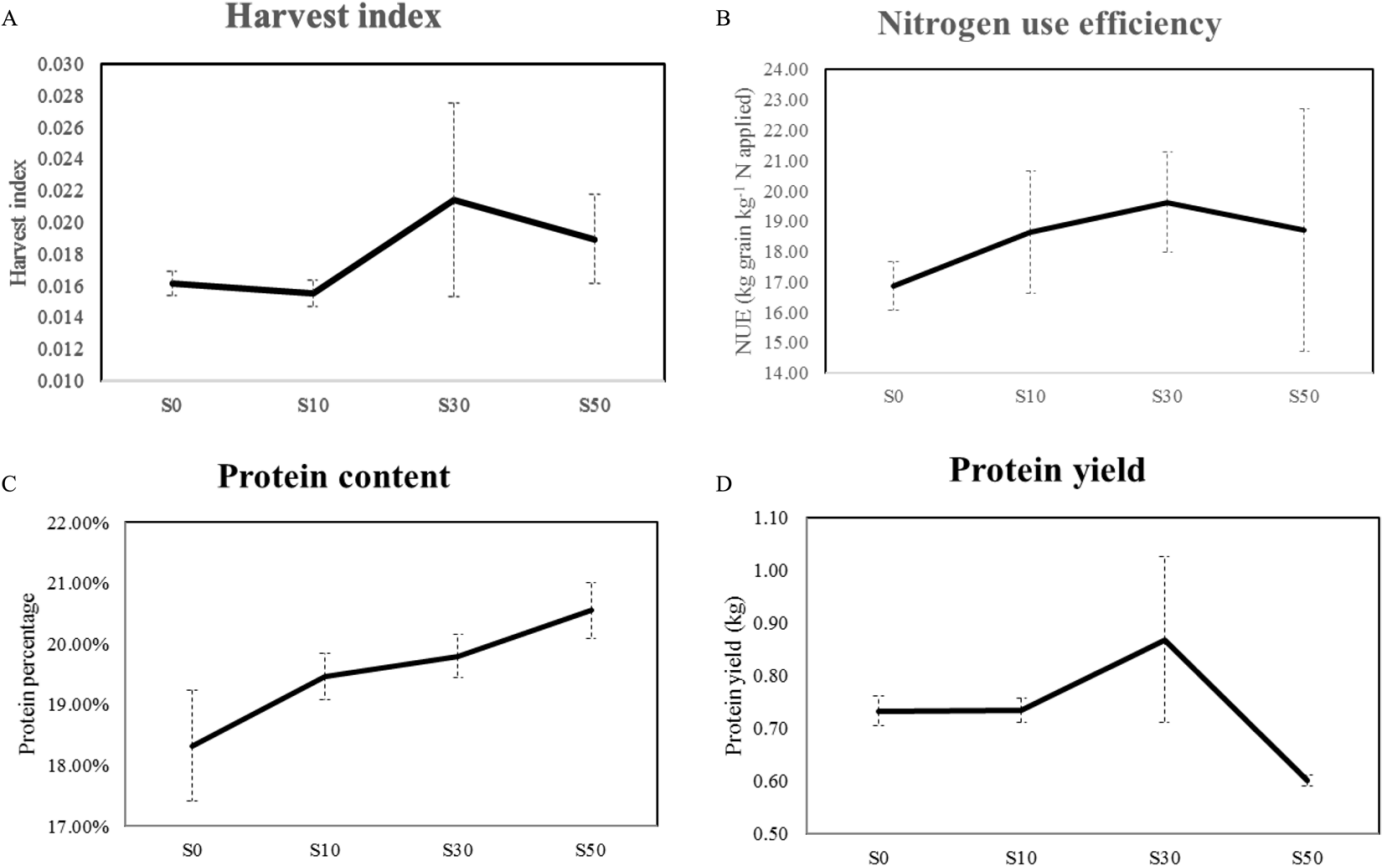
Impacts of sulphur on agronomic traits and protein parameters. (**A**) Harvest index. (**B**) NUE (kg grain yield generated by per hectare nitrogen applied). (**C**) Protein percentage. (**D**) Protein yield (protein percentage multiply by grain yield). Error bars were calculated from six biological replicates and one way ANOVA was used to test for significance at a *P* ≤ 0.05 level.

After seed germination in petri dishes, plants were transplanted into pots containing soil and fertilizer treatment. Both heading date and flowering time were tracked to determine the appropriate time of collection of developmental grain filling samples. Both biomass and grain yield were recorded after harvest, followed by calculation of harvest index (HI) and nitrogen use efficiency (NUE) for grain yield. Both HI and NUE were larger for treatment S30 than for S0; HI went from 0.016 to 0.021 and NUE from 16.86 kg to 19.63 kg, respectively. Conversely, both traits decreased when going from treatment S30 to S50; HI went from 0.021 to 0.019 (Fig. 1A) and NUE from 19.63 kg to 18.72 kg (Fig. 1B). There was a sharp increase in protein content, from 18.33% to 20.55% (Fig. 1C), between treatment S0 and S50, while protein yield first went up from 0.73 kg m^−2^ to 0.87 kg m^−2^ between treatments S0 and S30, followed by a decrease from 0.87 kg m^−2^ to 0.60 kg m^−2^ between treatments S30 and S50 (Fig. 1D).

**Figure 2:**
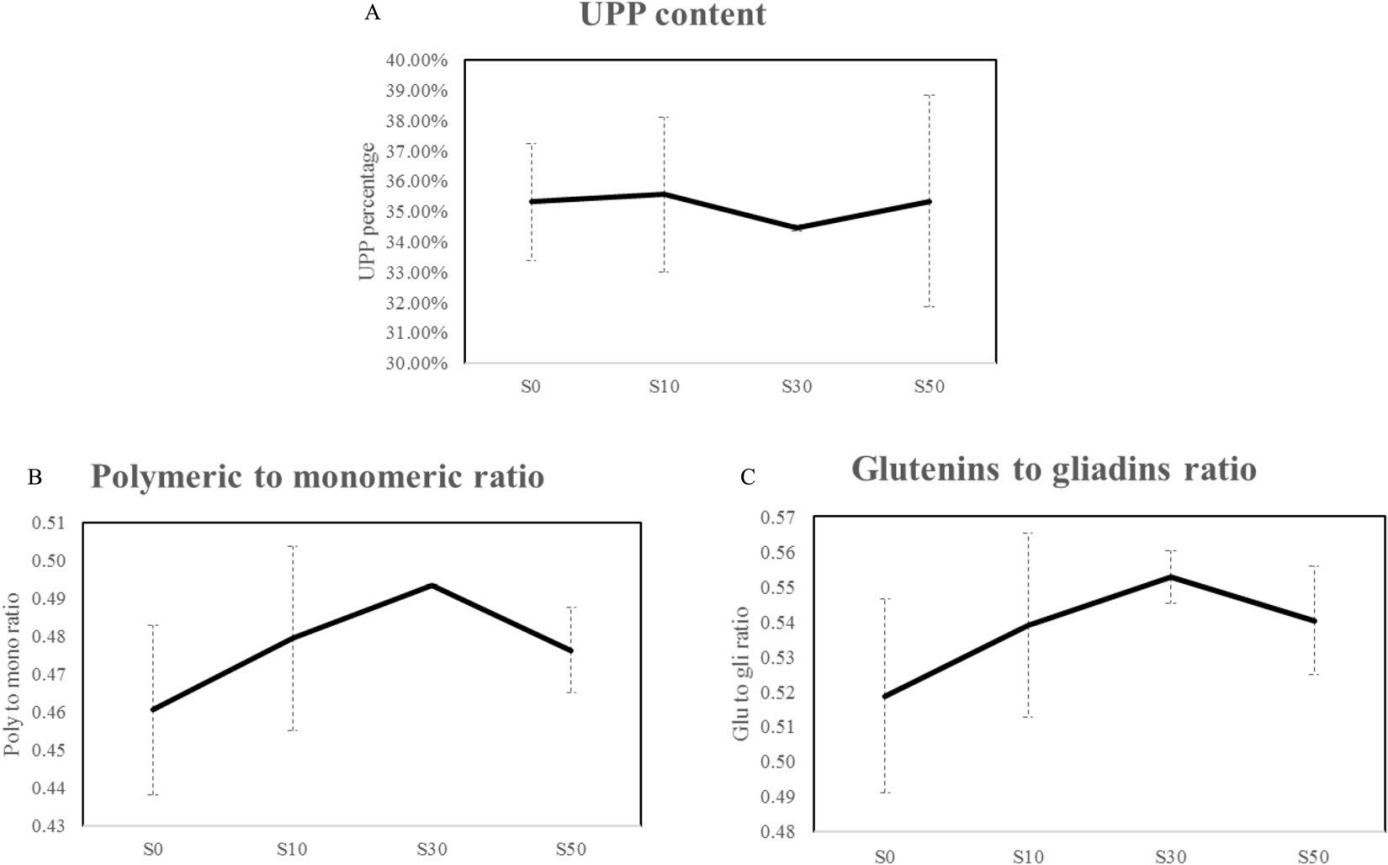
Impacts of sulphur on polymer and monomer. (**A**) The percentage of UPP. (**B**) The ratio of polymeric to monomeric proteins. (**C**) The ratio of glutenins to gliadins. Error bars were calculated from three biological replicates and one way ANOVA was used to test for significance at a *P* ≤ 0.05 level.

**Figure 3:**
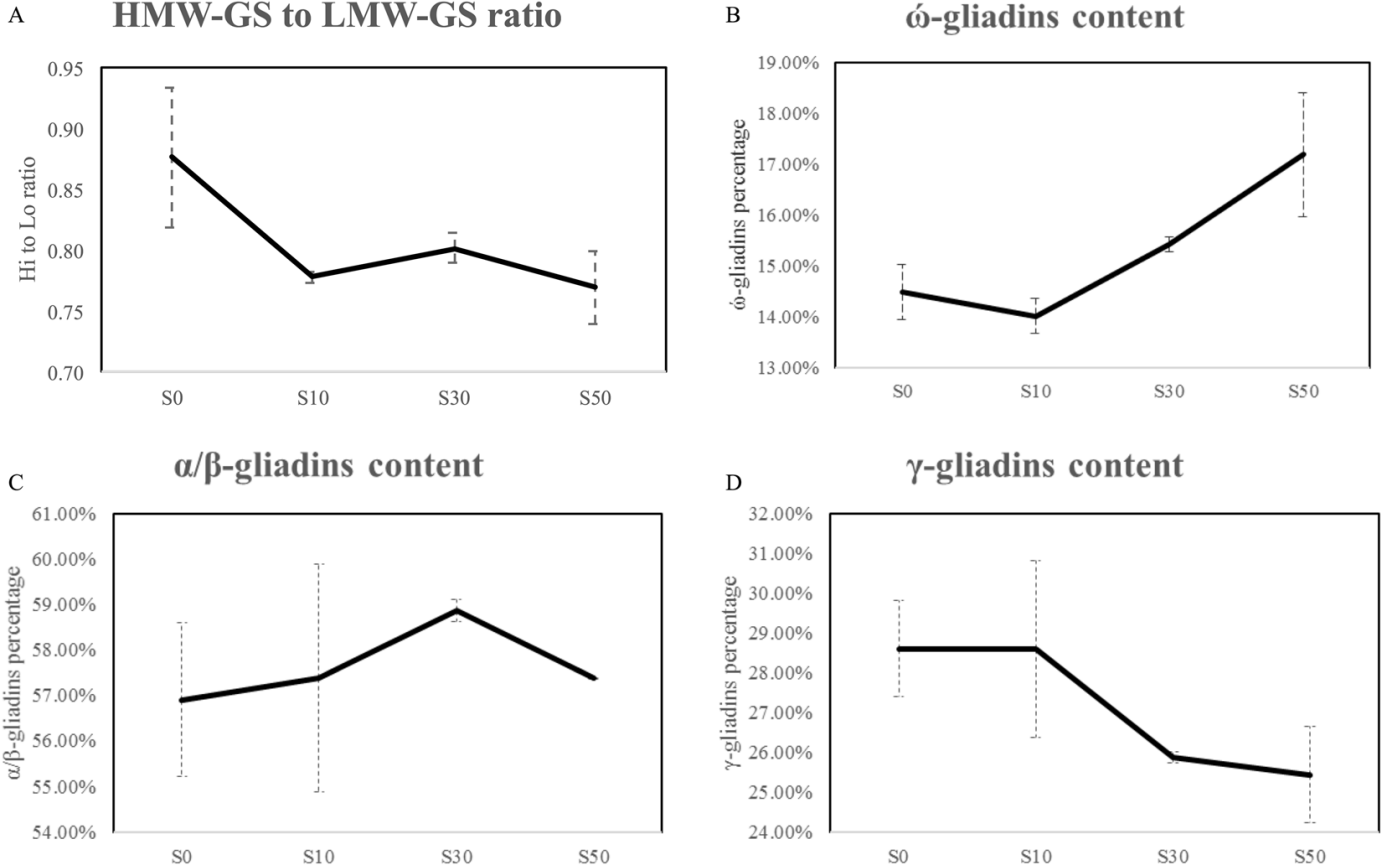
Impacts of sulphur on protein compositions. (**A**) The ratio of HMW-GS to LMW-GS. (**B**) The percentage of ώ-gliadins. (**C**) The percentage of α/β-gliadins. (**D**) The percentage of γ-gliadins. Error bars were calculated from six biological replicates and one way ANOVA was used to test for significance at a *P* ≤ 0.05 level.

UPP (SDS-unextractable polymeric proteins) content had a slight decrease with the amount of supplied sulphur from 0 to 30 kg ha^−1^, while a further increase in sulphur supply to 50 kg ha^−1^ increased the UPP content again (Fig. 2A). Both ratios of polymeric protein to monomeric protein and glutenins to gliadins were increased from 0.45 to 0.50 and 0.51 to 0.57 between treatments S0 and S30, but both decreased after raising the sulphur supply to 50 kg ha^−1^, moving from 0.50 to 0.47 and 0.57 to 0.53, respectively (Fig. 2B and C). The analysis of protein composition demonstrated that the ratio of HMW-GS (high molecular weight glutenin subunit) to LMW-GS (low molecular weight glutenin subunit) was apparently decreased between treatments S0 and S50 from 0.88 to 0.77 (Fig. 3A). However, an impact of sulphur on the modification of gliadins classes was not observed in the current study. With an increasing amount of sulphur from 0 to 50 kg ha^−1^, the percentage of ώ-gliadins increased from 14.50% to 17.19% (Fig. 3B), whereas γ-gliadins content decreased from 28.60% to 25.43% (Fig. 3D). The percentage of α/β-gliadins went from 56.90% to 58.87% between treatments S0 and S30, followed by a decrease to 57.37% after increasing supplied sulphur to 50 kg ha^−1^ (Fig. 3C).

### Transcriptome analysis

#### Differential expression genes (DEGs) and cluster of DEGs

After aligning 167,528,416 clean reads against International Wheat Genome Sequencing Consortium chromosome survey sequence, 127,222,144 total mapped reads were produced. The total mapped reads and multiple mapped reads of each sample were similar, at around 70% and less than 10%. The percent of reads mapped to exon regions of each sample was around 80%, while the reads density of each sample in chromosome 3B was higher than in other chromosomes. The expression level of genes was measured by transcript abundance. A higher the abundance corresponded to a higher the gene expression level. In this study, gene expression level was estimated by counting the reads mapped to exons. Read count was not only in proportion to the actual expression level of the genes, but also in proportion to the gene length and the sequencing depth. To make the estimated gene expression levels comparable across experiments, fragments per kilo base of transcript per million mapped reads (FPKM) was used for normalization of gene expression levels^36^. The FPKM method was capable of eliminating the influence of different gene lengths and sequencing discrepancies on the calculation of gene expression levels, and the calculation results can be used to make comparisons of differential gene expression levels among samples. In our study, differential expression analysis based on the padj set as less than 0.05 identified a total of 63 DEGs. When comparing treatments S30 and S0 at 7 DPA, we found that 62 DEGs were down-regulated, whereas only one DEG was up-regulated. The 63 DEGs were respectively located in 21 chromosomes except chromosome 3A. Due to the limited available annotation for wheat, there were 24 of 63 DEGs annotated with the use of *Triticum aestivum* as a reference organism. Among remaining 39 DEGs, 31 DEGs were annotated through the using of various plants as reference organisms such as *Arabidopsis thaliana* and *Oryza sativa*, while there was no available annotation for the remaining eight DEGs. Most of the 62 down-regulated DEGs were annotated as non-specific lipid-transfer proteins and enzymes involved in sulphur and nitrogen metabolic pathways, lipid biosynthesis and metabolic pathways, and glycolysis (Supplementary Table S1). The sole up-regulated DEG was annotated as a diphosphate-fructose-6-phosphate 1-phosphotransferase subunit alpha (PFP-ALPHA) involved in the biosynthesis of D-glyceraldehyde 3-phosphate and glycerone phosphate from D-glucose in the glycolytic pathway^37^ (Supplementary Table S1).

#### Functional annotation enrichment

**Figure 4:**
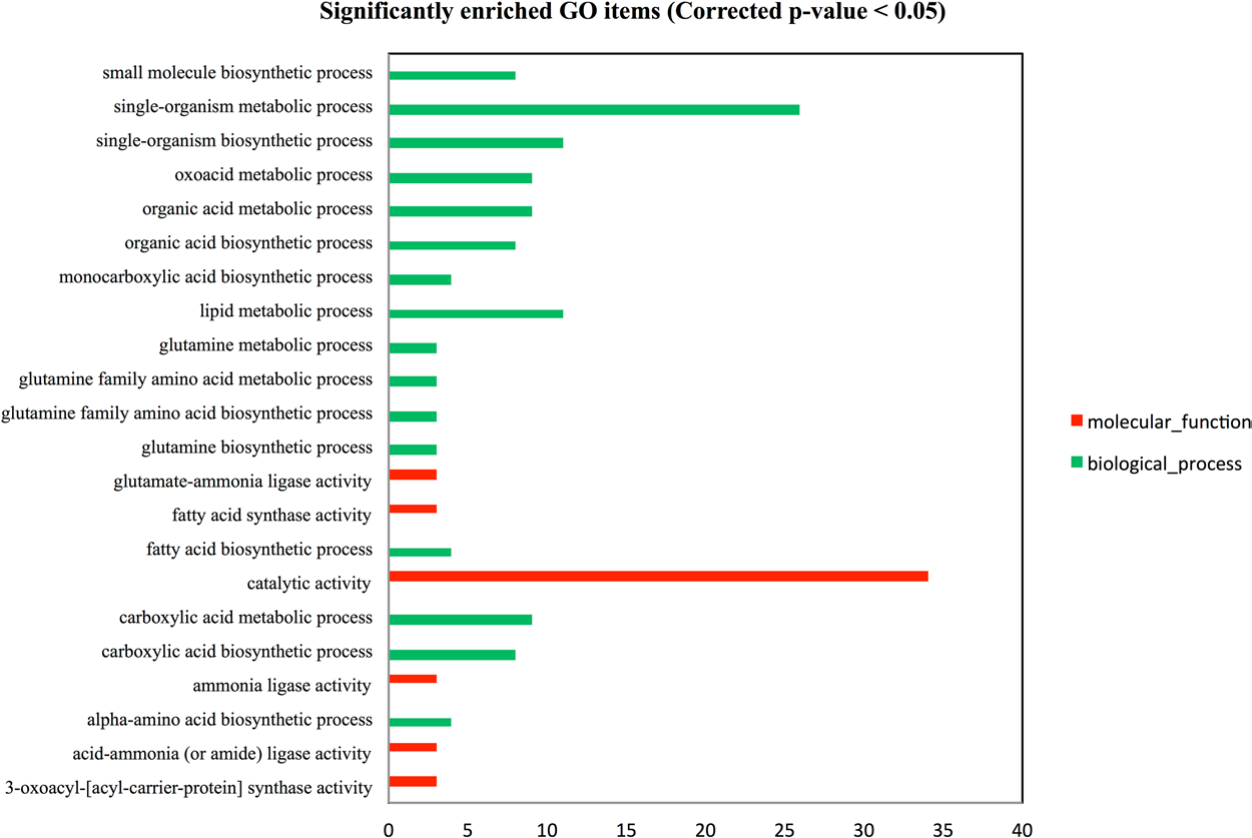
Significantly enriched GO items (corrected p-value ≤ 0.05). Biological process, green bar; Molecular function, red bar.

**Figure 5:**
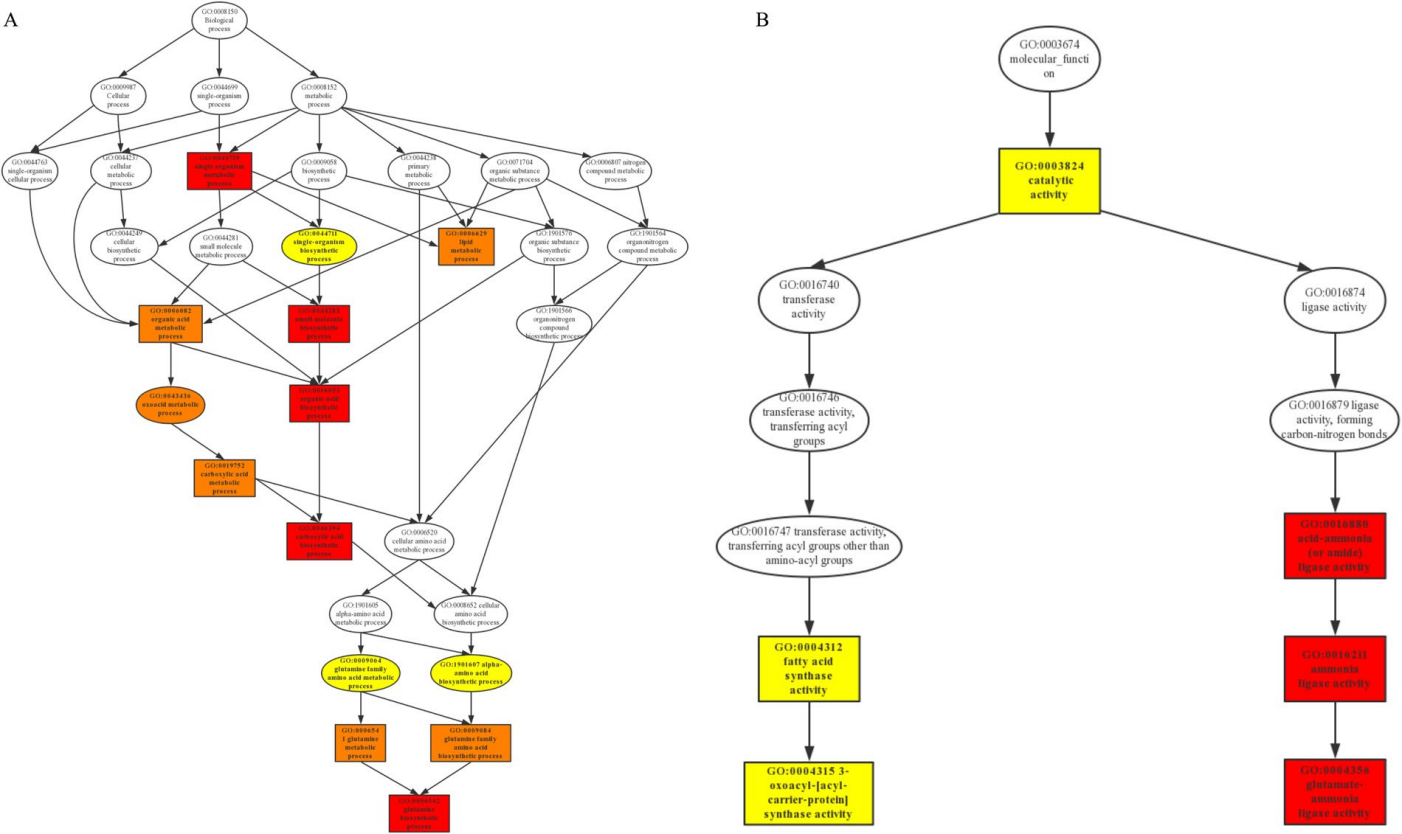
Directed acyclic graph of GO enrichment, DAG. The main nodes were shown by box. The enrichment degree was indicated by color shades, the darker the shades, the higher the enrichment degree. (**A**) Biological process. (**B**) Molecular function.

Three types of GO annotations were classified into biological process, molecular function and cellular component (Supplementary Table S2). The 63 DEGs were significantly enriched in 22 items with the corrected p-value ≤ 0.05 and all of 22 items were down-regulated by S30 at 7 DPA (Fig. 4). Among them, five items assigned to biological process and three items under molecular function were involved in nitrogen metabolism. The biological process is able to identify a set of molecular events with a defined beginning and end. There were three DEGs enriched in the processes of glutamine biosynthetic (GO: 0006542) and metabolic (GO: 0006541), and the processes of glutamine family amino acid biosynthetic (GO: 0009084) and metabolic (GO: 0009064), whilst there were four DEGs involved in the process of alpha amino acid biosynthesis (GO: 1901607). The molecular function describes the elemental activities of a gene product. The same three DEGs enriched in above biological processes were gathered in the activities of glutamate ammonia ligase (GO: 0004356), ammonia ligase (GO: 0016211) and acid-ammonia ligase (GO: 0016880). The cellular component type reflected the differing states of each tissue, while there was no significant GO enrichment in cellular component based on an FDR corrected p-value ≤ 0.05. Furthermore, the directed acyclic graphs (DAGs) of biological process and molecular function were used for the visualization of enriched GO items and their hierarchy, and illustrated the interaction and flow pathway of each item (Fig. 5A and B). The top ten significantly enriched items were selected as the main nodes and the darker colour indicated higher DEG enrichment. The DAG of biological process demonstrated that DEGs were significantly enriched in the GO items annotated as nitrogen metabolic relevant mechanisms, which were at the bottom of biological process hierarchy. Both glutamine family amino acid metabolic and alpha amino acid biosynthetic pathways were capable of regulating glutamine metabolic and glutamine family amino acid biosynthetic pathways, whose function was to regulate the glutamine biosynthetic process. The DAG of molecular function illustrated that one cluster was focused on fatty acid metabolic pathways, while another was concentrated on glutamine metabolism. DEGs enrichment was more significant in glutamine than in fatty acid metabolism. The DEGs enrichment analysis revealed that there were four DEGs enriched in nitrogen metabolic pathways. Three of four DEGs (*Traes_6 AL_2017727C4*, *Traes_6BL_95C7F7123* and *Traes_6DL_24A8AB125*) were annotated as glutamine synthetase in hexaploid wheat, while the rest one DEG (*Traes_7AL_9AEC84938*) was identified as UPF0481 protein At3g47200 in Arabidopsis annotation database (Table 1).

**Table 1:** Identification of three DEGs annotated as glutamine synthetase.

Up/down-regulation (S30 vs. S0)	Gene ID	Gene orthology annotation	Swiss-Prot
Down-regulation	Traes_6AL_2017727C4	Molecular function GO:0016880 acid ammonia (or amide) ligase activity GO:0016211 ammonia ligase activity GO:0004356 glutamate ammonia ligase activity Biological process GO:0009064 glutamine family amino acid metabolic process GO:1901607 alpha amino acid biosynthetic process GO:0006541 glutamine metabolic process GO:0009084 glutamine family amino acid biosynthetic process GO:0006542 glutamine biosynthetic process	Glutamine synthetase
	Traes_6BL_95C7F7123		Glutamine synthetase
	Traes_6DL_24A8AB125		Glutamine synthetase

#### KEGG pathway enrichment

**Figure 6:**
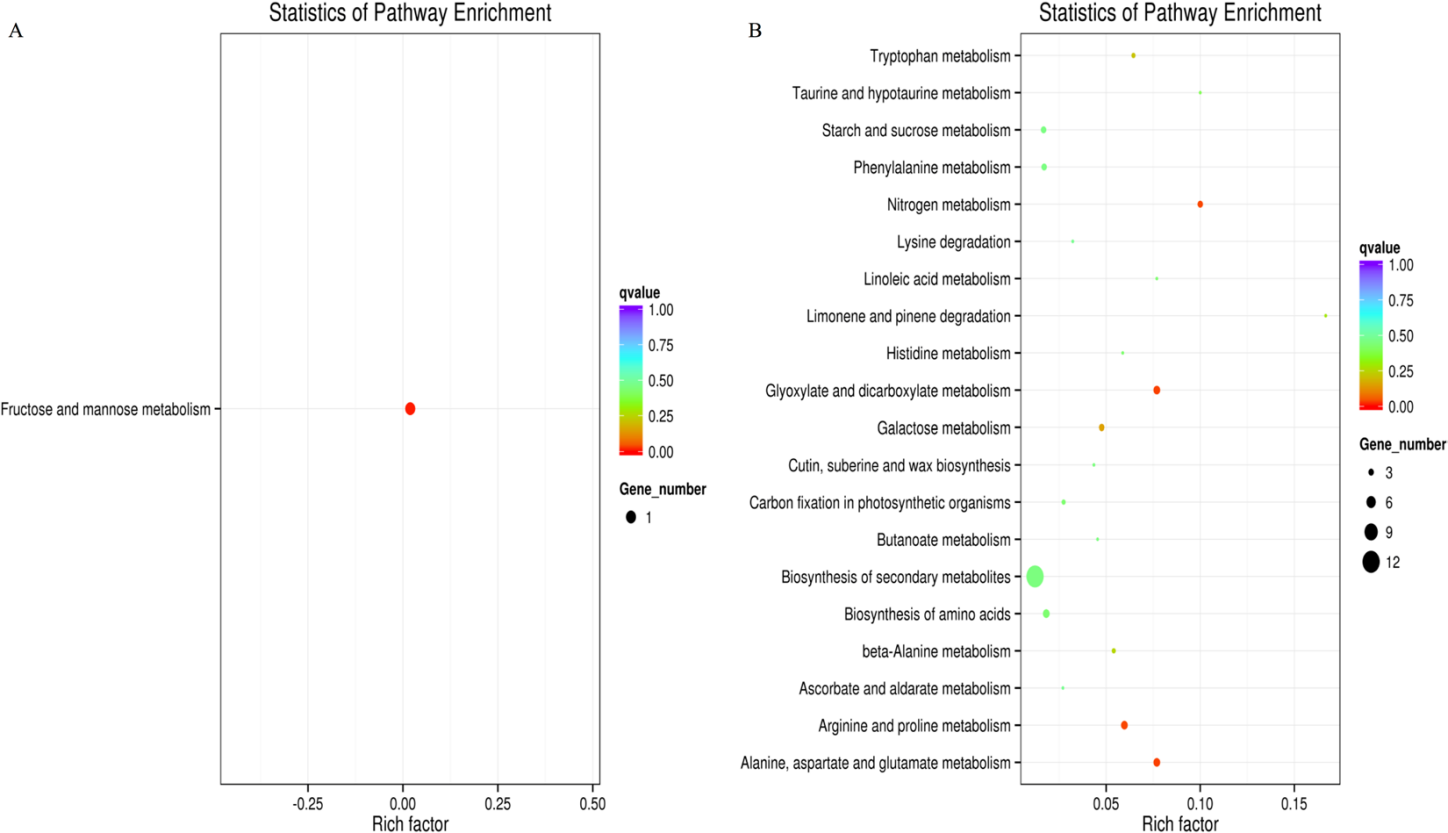
KEGG pathway enrichment scatter plot. (**A**) Up-regulated pathway. (**B**) Down-regulated pathway.

The interactions of DEGs were involved in certain biological functions. KEGG pathway enrichment analysis identified significantly enriched metabolic pathways and signal transduction pathways associated with DEGs when compared with the whole genome background (Supplementary Table S3). The top 20 most significantly enriched pathways were selected to produce the KEGG scatter plot. The enrichment degree of a pathway was determined by using the rich factor, qvalue and gene count enriched to pathway ratio. The rich factor was the ratio of the number of DEGs to the number of genes annotated in a given pathway. The qvalue was the p-value after normalization. A pathway with a larger rich factor was indicative of higher enrichment, and a pathway with a qvalue closer to zero indicated a more significant enrichment. The statistics of pathway enrichment analysis revealed that only fructose and mannose metabolism pathway (bdi: 100833293) was up-regulated by S30 (Fig. 6A), whereas other 20 pathways were down-regulated by high sulphur treatment (Fig. 6B). Among them, DEGs were more significantly enriched in two pathways named as arginine and proline metabolism (bdi: 100845598) and alanine, aspartate and glutamate metabolism (bdi: 100845598) than other 17 metabolism pathways, but except glyoxylate and dicarboxylate metabolism pathway (bdi: 100845598). Three DEGs (*Traes_6AL_2017727C4*, *Traes_6BL_95C7F7123* and *Traes_6DL_24A8AB125*) annotated as glutamine synthetase (100845598) were enriched in above two metabolic pathways. Beside these, *Traes_3DS_E756029E7* located on chromosome 3DS was enriched in alanine, aspartate and glutamate metabolism pathway and *Traes_2DL_04892661A* located on chromosome 2DL was enriched in arginine and proline metabolism pathway. Beside these, the sole up-regulated DEG *Traes_5BS_B4326E4BD* located on chromosome 5BS was enriched in fructose and mannose metabolic pathway (Table 2).

**Table 2:** DEGs significantly enriched KEGG pathways (corrected p-value ≤ 0.05).

Up/down-regulation (S30 vs. S0)	Term	ID	Input DEGs	KEGG_ID/KO
Down-regulation	Alanine, aspartate and glutamate metabolism	bdi00250	*Traes_6AL_2017727C4* *Traes_6BL_95C7F7123* *Traes_6DL_24A8AB125* *Traes_3DS_E756029E7*	bdi:100845598 bdi:100845598 bdi:100845598 bdi:100827187
	Nitrogen metabolism	bdi00910	*Traes_6AL_2017727C4* *Traes_6BL_95C7F7123* *Traes_6DL_24A8AB125*	bdi:100845598 bdi:100845598 bdi:100845598
	Arginine and proline metabolism	bdi00330	*Traes_6AL_2017727C4* *Traes_6BL_95C7F7123* *Traes_6DL_24A8AB125* *Traes_2DL_04892661A*	bdi:100845598 bdi:100845598 bdi:100845598 bdi:100824147
	Glyoxylate and dicarboxylate metabolism	bdi00630	*Traes_6AL_2017727C4* *Traes_6BL_95C7F7123* *Traes_6DL_24A8AB125* *Traes_6DS_3522B8EF6*	bdi:100845598 bdi:100845598 bdi:100845598 bdi:100846426
Up-regulation	Fructose and mannose metabolism	bdi00051	*Traes_5BS_B4326E4BD*	bdi:100833293

#### Real time PCR (RT-PCR) validation

The transcriptomic study shows that glutamine synthetase (GS) as a pivotal enzyme in GOGAT cycle for nitrogen metabolism is regulated by sulphur availability. This is a bridge of sulphur and nitrogen interaction. Therefore, RT-PCR analysis on *GS* was conducted to validate the results of RNA-seq.

**Figure 7:**
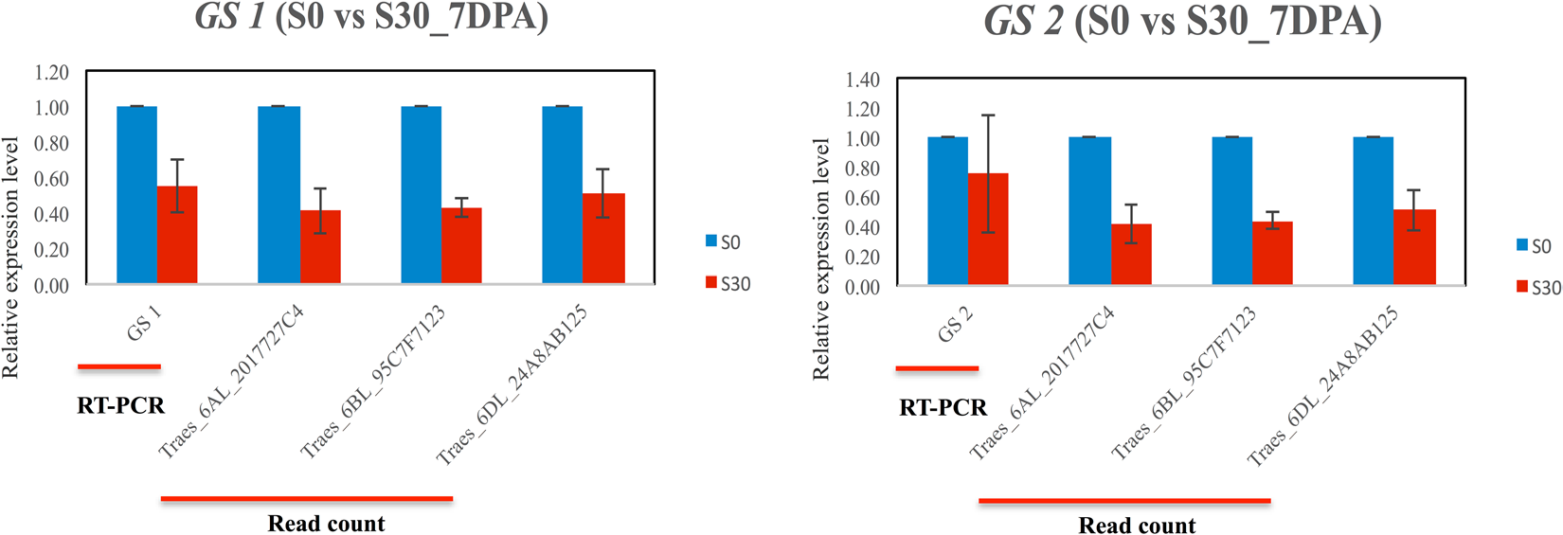
Real time PCR validation. (**A**) the expression level of *GS1* and three DEGs in two treatments at 7 DPA. (**B**) the expression level of *GS2* and three DEGs in two treatments at 7 DPA.

As mentioned above, the *GS1* is located on chromosome 6A, while the *GS2* is located on chromosome 5D. Therefore, three significant enriched DEGs annotated as glutamine synthetase and located on chromosome 6 are speculated to be *GS1*. Sequence alignment showed that the three DEGs are nearly the same as *GS1* except few SNPs among them plus a 5-bp insertion in *Traes_6AL_2017727C4* (Supplementary Fig. S1a). In comparison, more variations were found between the three DEGs and *GS2* (Supplementary Fig. S1b). After all, the RT-PCR results clearly demonstrated that both *GS1* and *GS2* are downregulated by high sulphur treatment (S30) in 7 DPA, which is in accordance with the RNA-seq results of the three DEGs (Fig. 7).

#### Promoter motif analysis

**Figure 8:**
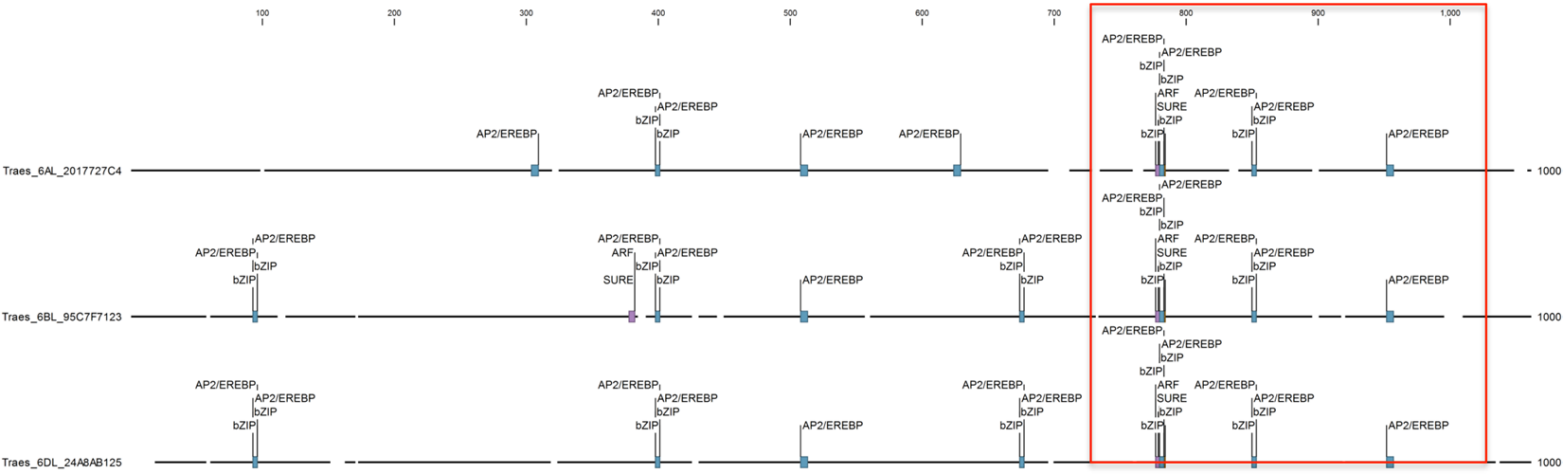
*Cis*-acting element conserved regions in promoter sequence of three DEGs annotated as glutamine synthetase.

Conserved regions for *trans*-acting elements binding were found in the 700 to 1000 bp promoter regions of three DEGs (*Traes_6AL_2017727C4*, *Traes_6BL_95C7F7123* and *Traes_6DL_24A8AB125*) annotated as glutamine synthetase. The binding sites for transcription factor AP2/EREBP, bZIP, ARF and SURE, located in 700 to 1000-bp upstream region, were highly conserved (Supplementary Table S4). Analysis of phytohormone specific transcription factors illustrated that *cis*-acting elements for ethylene and auxin were highly conserved in this region as well (Fig. 8).

## Discussion

Our analysis of agronomic traits after various combinations of sulphur and nitrogen fertilizer treatments showed that both HI and NUE increased along with increasing sulphur supply to 30 kg ha^−1^. Analysis of protein parameters showed that protein content and yield both increased with increasing sulphur supply to 30 kg ha^−1^ as well. However, even though both HMW-GS and LMW-GS content increased, the ratio of HMW-GS to LMW-GS decreased, suggesting that the latter increased to a larger extent. We didn’t observe any impact on the composition of the gliadin fraction caused by varying sulphur supply. Moreover, the content of UPP relative to total protein is the major determinant of rheological properties of dough. The amount of UPP in flour (around 20 to 40 mg g^−1^) is strongly correlated with dough strength and loaf volume, and together with the polymeric to monomeric proteins ratio, constitutes a quantitative factor for dough quality. The molecular weight distribution (MWD) of glutenins has been recognized as one of the main determinants of physical dough properties^38,39^. The results of the present study showed that an increase in sulphur supply did not cause a significant change of UPP content. However, it is worth noting that no significant changes in the gliadin biosynthesis was observed for each treatment while the reduction in the HMW-GS to LMW-GS ratio under increased sulphur supply was obvious (Fig. 3A), which implies that sulphur deficit was happening during grain-filling. The impact of sulphur deficiency on wheat correlates with its availability. A moderate sulphur deficiency mostly affects protein quality rather than yield, while severe sulphur deficiency affects yield. In the current study sulphur was applied at the beginning of seed germination rather than at flowering or grain-filling stage, which suggests that sulphur deficiency occurs due to unspecific mechanisms of sulphur transporters preventing the improvement of sulphur use efficiency (SUE). The later the deficiency takes place, the less it affects sulphur content in the seeds. Therefore, the above analysis suggested that mid-season sulphur deficiency had occurred in this study.

One sulphur deficiency symptom is the yellowing of new leaves, in contrast to nitrogen deficiency which affects old leaves first. All sulphur delivered to the grain must be released via remobilization from other tissues. Therefore, the translocation and remobilization of sulphur and its delivery to seed are of great importance for SUE^40^. Differential expression analysis reported in the literature assumes that sulphate transporters play a role in the control of sulphate fluxes throughout the entire process of plant development. There are four sulphate transporter gene families in wheat and each of them has specific roles to play. Three genes in group one are annotated as plasma membrane located high-affinity sulphate transporters, which are induced by sulphate deprivation in roots. Only one gene assigned to group two is a low-affinity transporter and expressed in vascular tissues. There are five genes belonging to group three, while their functions are vague at present. The last gene in group four is involved in vacuolar efflux^41,42^. Among all these sulphate transporters, *SULTR1;1* in group one was annotated as a high-affinity sulphate transporter and significantly up-regulated under sulphur deficiency. The 16 bp sulphur responsive element (SURE) of *SULTR1;1* promoter was reported as a positive sulphur deficit responsive *cis*-acting element without any additional elements required for its fundamental function. The 5 bp core sequence (GAGAC) acts as a core element for sulphur deficiency response of SURE and may commonly regulate the expression of genes required for adaption to sulphur deficit^7^. Therefore, the three DEGs annotated as glutamine synthetase were down-regulated under high sulphur supply and the presence of the SURE core sequence in the promoter region demonstrated that sulphur deficiency happened at low sulphur treatment (Fig. 4).

Plants have the ability to store sulphur to deal with short term sulphur deficiency. For instance, plants can activate sulphate uptake and primary sulphur assimilation for cysteine synthesis. The interorgan transport of sulphate may be regulated for efficient distribution and utilizes the internal vacuolar reserve. In addition, secondary sulphur metabolites can be remobilized as an additional source of sulphur for primary assimilation. However, long-term sulphur starvation results in a decrease in the level of total proteins, chlorophyll, RNA, and biomass, with the concomitant adverse impacts on plant development and the accumulation of protein^43–45^.

HI and NUE are two crucial agronomic traits for the enhancement of yield, and various protein parameters are a concern for the improvement of various end-products quality. Additionally, the regulatory interaction between sulphate assimilation and nitrate reduction is believed to occur at the transcriptional level. In this study, significant difference of HI, NUE, and various protein parameters were observed between S0 and S30, and were therefore subsequently selected for transcriptomic study to explore the potential mechanism.

According to a model proposed by Lewandowska & Sirko^26^ in 2008, a plant’s response to sulphur starvation can be divided into three major stages and depends on the degree and duration of starvation. During the initial stage, changes happen to the expression of primary genes in the sulphur assimilation pathway and sulphate uptake from soil, followed by remobilization of stored inorganic sulphur from the vacuole. However, if sulphur remains a limiting factor, changes will occur in multiple metabolic pathways. Plants intensify organic sulphur fluxes and activate stress defence responses, followed by the down-regulation of genes responsible for the uptake and assimilation of nitrogen. Changes in plant developmental processes result in an increase of root to shoot biomass ratio and the earlier activation of senescence mechanisms. However, both root and shoot growths are slowed down and the reproductive phase kicks in earlier in order to save sulphur for grain filling^46^.

Under sulphur deficiency conditions, the ability of plants to take up nitrate and ammonium diminishes while sulphur uptake ability increases. Due to the reduction of sulphur containing amino acids such as cysteine and methionine, protein biosynthesis is blocked, leading to the accumulation of both inorganic and organic nitrogenous compounds such as arginine and asparagine^47,48^. Free asparagine can promote the formation of acrylamide by reacting with reducing sugars via the Maillard reaction during high-temperature bread-making processes. Acrylamide is a suspected food carcinogen^49,50^.

As mentioned above, one nutrient may accumulate when another is limiting. The accumulated nutrient can then be used in protein synthesis when the deficit is overcome in the short term. Therefore, longer sulphur starvation results in a decrease in the level of protein, which leads to the accumulation of glutamine as a nitrogen store. The present transcriptomics study demonstrated that three DEGs annotated as glutamine synthetase (NADH-GOGAT) and located on chromosomes 6AL, 6BL and 6DL were down-regulated under high sulphur supply. In addition, the KEGG pathway analysis indicated that three pathways involved in nitrogen and amino acid metabolism were down-regulated under the high sulphur treatment. These results revealed that there was less accumulation of free stored nitrogenous compounds when sufficient sulphur was supplied. Moreover, under low sulphur condition, sulphur starvation results in enhanced GS activity, which leads to the accumulation of asparagine due to the blockage of protein biosynthesis, thus promoting the formation of acrylamide by free asparagine and reduced sugars during the bread-making process, which could potentially increase the risk of cancer development in humans. Therefore, the application of fertilizer to achieve a sufficient supply of sulphur is an important consideration in wheat crop management in order to maximise yield and end-product quality.

## Material and Methods

### Plant material, growth conditions and sample collection

Plants of hexaploid wheat cultivar Spitfire were transplanted after vernalisation into 24.4 L cubic pots filled with low sulphur containing soil delivered from Kataning agricultural research station of Department of Agriculture and Food, Western Australia (DAFWA). The experimental treatments consisted of four ranges of sulphur supplement, which were 0 kg ha^−1^, 10 kg ha^−1^, 30 kg ha^−1^ and 50 kg ha^−1^, expressed as S0, S10, S30 and S50. There were six biological replicates for each combination. Gypsum (18% S) was used as the sources of sulphate. Urea (46% N), triple superphosphate (20.5% P) and muriate of potash (50% K) was respectively used as nitrate (N), phosphorus (P) and potassium (K) source at 50 kg ha^−1^, 60 kg ha^−1^ and 100 kg ha^−1^. Fertilizer were mixed with soil before transplanting the plants into pots and the amount of gypsum and urea for each type of combination was calculated according to the treatment design. The pots were placed in a glasshouse and arranged in a randomized complete block design (RCBD). Growth conditions in the glasshouse were 20 °C/11 °C (day/night) for an 8 hr light and 16 hr dark photoperiod. Soil moisture was adjusted to 70% field capacity. Each pot was watered every morning with demineralized water.

Both heading date and flowering time were recorded. Main stem grain was targeted and collected at 7 day intervals, with sample collection starting at seven days post-anthesis (7 DPA) and continued till 42 DPA. Two or three grains of the central stem were taken and immediately frozen in liquid nitrogen and then stored at −80 °C for RNA extraction. Lastly, mature grain was harvested and weighed to determine grain yield and plant biomass for each pot. HI was calculated as the ratio of grain yield to biomass and NUE was computed as grain yield per kilogram of nitrogen applied (kg ha^−1^). Protein content for each sample was measured using near-infrared spectroscopy (NIR) according to CSIRO methodology^51^ and protein yield was calculated as protein percentage multiplied by grain yield (kg m^−2^).

### Protein extraction

#### Sequential extraction of SDS-extractable polymeric protein (EPP) and SDS-unextractable polymeric protein (UPP)

EPP and UPP extraction was performed according to Batey *et al*.^52^. Generally, 100 mg of grain was ground into wholemeal flour using TissueLyser II from Qiagen. 1 ml of 0.05 M phosphate-buffered saline pH 6.9 (PBS, HPLC grade) buffer with 0.05% sodium dodecyl sulphate (SDS) was added to the flour without sonication for the extraction of EPP. After removing the supernatant, another 1 ml of 0.05 M 0.05% SDS PBS extraction buffer pH 6.9 was added to the precipitate for the extraction of UPP. The pellet was suspended in the solution and sonicated for 1 min at a 300 W power setting (15 rounds of 2 sec pulses and 2 sec intervals). Afterwards, the supernatant was collected as UPP. Finally, both EPP and UPP extracts were filtered using a 0.45 µm filter.

#### Sequential extraction of gliadins and glutenins

Glutenins were extracted according to Yu *et al*.^53^. Briefly, 100 mg grain was ground into wholemeal flour using TissueLyser II from Qiagen. This was followed by the addition of 70% ethanol to the flour for the extraction of gliadins, and then 55% isopropanol was added to the precipitate to remove the albumin and globulin fractions. Subsequently, 1 M Tris-HCl pH 8.0 and 1% dithiothreitol (DTT) were used to disrupt disulphide bonds, followed by 1.4% 4-vinylpyridine (4-VP) to prevent the formation of reductive disulphide bonds. Lastly, glutenins were precipitated with 60% of cold acetone. After purifying the extract with 100% ethanol and acetone containing 0.07% β-mercaptoethanol (β-ME), the final extract was kept in a solution containing 0.05% trifluoroacetic acid (TFA) and 50% acetonitrile (ACN).

### Separation and quantification of EPP and UPP, gliadins and glutenin subunits

#### Size-exclusion high performance liquid chromatograph (SE-HPLC)

The separation and quantification of EPP and UPP was performed by HPLC using an Agilent 1200 LC system (Agilent Technologies, http://www.agilent.com). 10 µl of the extracts were injected into a Bio SEC-5 (4.6 × 300 mm, 500 Å, Agilent Technologies) column maintained at room temperature. The eluents used were ultrapure water (solvent A) and acetonitrile (solvent B), each containing 0.1% TFA (HPLC grade, Sigma Aldrich). The flow rate was adjusted to 0.35 ml min^−1^. Protein was separated by using a constant gradient with 50% of solvent A and 50% of solvent B in 15 mins and detected by UV absorbance at 214 nm. Each sample was sequentially injected twice for technical replication. After the runs, the column was washed with 50% of ultra-pure water (solvent C) and 50% of methanol (Solvent D) applied at a 0.2 ml min^−1^ flow rate. Both acetonitrile and methanol used as eluents were HPLC grade (Fisher Scientific).

#### Reverse-phase high performance liquid chromatograph (RP-HPLC)

The separation and quantification of gliadins classes and glutenin subunits were performed by HPLC using an Agilent 1200 LC system (Agilent Technologies, http://www.agilent.com). 10-µl of extract was injected into a C18 reversed-phase Zorbax 300 StableBond column (4.6 × 250 mm, 5 µm, 300 Å, Agilent Technologies) maintained at 60 °C. The eluents used were ultrapure water (solvent A) and acetonitrile (solvent B), each containing 0.06% TFA (HPLC grade, Sigma Aldrich). The flow rate was adjusted to 0.6 ml min^−1^. Protein was separated by using a linear gradient from 21% to 47% of solvent B in 45 mins and detected by UV absorbance at 214 nm. 15 mins post-run was used for column balance after every sample run. Every sample was sequentially injected twice for technical replication. After finishing the runs, the column was washed with 50% of ultrapure water (solvent C) and 50% of methanol (Solvent D) using a 0.5 ml min^−1^ flow rate. Both acetonitrile and methanol used for eluents were HPLC grade (Fisher Scientific).

Chromatograms were processed using ChemStation for LC 3D systems software (Revision B.03.02 [341], Agilent Technologies). Four HPLC peaks (P1, P2, P3 and P4) corresponding to each EPP and UPP component were identified as glutenins (P1), gliadins (P2 + P3), and albumins and globulins (P4) following the observations of Johansson *et al*.^54^ and Sissons *et al*.^55^. Calculation of the ratio of polymeric to monomeric (P/M), ratio of glutenins to gliadins (glu/gli), and the percentage of UPP (UPP %) was performed using the area-under-the-peak method. Thus, P/M = (P1 + P1 after sonication)/(P2 + P3 + P4 + P2 after sonication + P3 after sonication + P4 after sonication); glu/gli = (P1 + P1 after sonication)/(P2 + P2 after sonication); UPP % = P1 after sonication/(P1 + P1 after sonication).

Three sequential boundary areas corresponding to four classes of gliadins were regarded as ω-gliadins, α/β-gliadins, and γ-gliadins, and two obvious boundary areas in the glutenins chromatogram corresponded to HMW-GS and LMW-GS based on the hydrophobicity of each protein^56^. The amount of each gliadin class and glutenin subunit was calculated as the percentage of total gliadins and glutenins divided by the total area under the chromatogram trace.

### RNA isolation, library construction and sequencing

Grain from three biological replicates was ground in liquid nitrogen and total RNA was extracted using pre-chilled Trizol reagent (Invitrogen, Carlsbad, CA) following the manufacturer’s directions, with some modifications. Protein was removed with protein extraction buffer (1 M Tris-HCl, 5 M NaCl, 10% SDS, 0.125 M EDTA, and 1 M DTT). After protein extraction the acid phenol/chloroform/isopropanol (49:49:2), Trizol and chloroform were added sequentially for the extraction of total RNA. Isopropanol was used for the precipitation of total RNA, which was subsequently treated with the Qiagen DNase kit to remove potential genomic DNA contamination. Concentration and purity were checked by Nanodrop, with 260/280 absorbance ratios of approximately 2.0, and the degradation and potential contamination was tested by agarose gel electrophoresis. RNA integrity was confirmed with an Agilent 2100 Bioanalyzer (Agilent Technologies, Palo Alto, CA).

The mRNA was enriched using oligo (dT) beads and then fragmented randomly in fragmentation buffer, followed by cDNA synthesis using random hexamers and reverse transcriptase. After first-strand synthesis, a custom second-strand synthesis buffer (Illumina) was added together with dNTPs, RNase H and *Escherichia coli* polymerase I to generate the second strand by nick-translation. The final cDNA library was ready after a round of purification, terminal repair, A-tailing, ligation of sequencing adapters, size selection and PCR enrichment. Library concentration was first estimated using a Qubit 2.0 fluorometer (Life Technologies), and then diluted to 1 ng μl^−1^ before checking insert size on an Agilent 2100 Bioanalyzer. The concentration was then quantified at greater accuracy by quantitative PCR (Q-PCR) (library activity >2 nM). Each library with an individual barcode was sequenced by Illumina HiSeq^TM^ PE125/PE150 (Illumina Inc., USA).

### Transcriptomic analysis

#### Quality control

After initial data quality control, the sequence datasets for six samples were pooled in six data files, in total consisting of 174,143,328 raw reads. Afterwards, raw reads were filtered to remove reads containing adaptor contamination; reads containing N more than 10%; and reads with more than 50% low-quality nucleotides (base quality less than 20). In total, 6,614,912 reads were removed, and the analysis was performed on the remaining 167,528,416 clean reads. The retained clean reads were pair-end mapped to the International Wheat Genome Sequencing Consortium chromosome survey sequence of Chinese Spring using HISAT 0.1.5 beta (Hierarchical Indexing for Spliced Alignment of Transcripts, release 25 February 2015). For the analysis, the mismatch parameter was set as at most two nucleic acids, and other parameters were set as default. Total mapped reads (TMR) of more than 70% and multiple mapper reads (MMR) of less than 10% were used as standard for the verification of mapping quality.

#### Differentially expressed genes (DEGs) analysis

For the fragment per kilo base of transcript per million mapped reads (FPKM) a value of 1.0 was set as the threshold for determining whether a gene was expressed or not. HiSeq v0.6.1 (A Python package for high-throughput sequencing data analysis) was used to analyse gene expression levels in this experiment, using the union mode. The correlation between samples was justified by the square of the Pearson correlation coefficient. The DESeq (version 1.10.1, R Bioconductor package) was used to conduct the differential expression analysis. The normalized data were fitted to a negative binomial generalized linear model. The threshold of the p-value after normalization (padj, qvalue) was set as ≤0.05 for filtering accurate DEGs. The clustering of DEGs was analysed based on FPKM value with the use of ggplot2 (version 2.1.0) and pheatmap (version 1.0.8).

Gene ontology (GO) and Kyoto Encyclopedia of Genes and Genomes (KEGG) pathway enrichment analysis of DEGs:GOseq (R Bioconductor package) based on Wallenius non-central hyper-geometric distribution was used for Gene ontology (GO, http://geneontology.org/) enrichment analysis^57^. GO with false discovery rate (FDR) corrected p-value ≤ 0.05 was regarded as significant enrichment. KOBAS (version 2.0, http://kobas.cbi.pku.edu.cn/), a web server for annotation and identification of enriched pathways and diseases, was applied for Kyoto Encyclopedia of Genes and Genomes (KEGG, http://www.genome.jp/kegg/) pathway enrichment analysis. The formula for the pathway enrichment analysis was

$$p=1-\sum _{i=0}^{m-1}\frac{(\begin{array}{cc}M & N-M\\ i & n-i\end{array})}{(\begin{array}{c}N\\ n\end{array})}$$

(N, the number of all genes with a KEGG annotation; n, the number of DEGs in N; M, the number of all genes annotated to specific pathways; m, number of DEGs in M). Pathways with FDR corrected p-value ≤ 0.05 were considered as significant enrichment.

#### Real time PCR

First strand cDNA was synthesized based on the manufacturer of Revert Aid First Strand cDNA Synthesis Kit (Thermo Fisher Scientific, USA). 5 μg DNase-treated RNA and random primers were used. The expression levels of *GS1* and *GS2* in three biological replicates for two treatments were quantified by real time PCR with SYBR-green as the intercalated dye (Qiagen Rotor-Gene Q instrument), and the 2^−ΔΔCT^ method. The primers for *GS1* and *GS2* were designed using Primer Premier 5.0 based on their respective specific coding regions (Supplementary Table. S5). The amplified efficiency of each primer pair was testified, and the melting curve of each primer pair demonstrated a single peak. ADP-ribosylation factor and Actin 3 were selected as reference genes, and the PCR was performed in a 20 μL reaction volume with 4 pM of each primer, 1 μL of the first-strand cDNA and 1× SYBR Premix Ex Taqۛ Π (Takara, Kyoto, Japan). The amplification reactions were carried out with the initial denaturing temperature of 94 °C for 10 mins, followed by 40 cycles of 94 °C for 10 secs, annealing temperatures of each primer 15 secs and the extension at 72 °C for 30 secs. The relative expression level of each gene was the means of three biological replications ±SD, and three technical replications were conducted for each gene.

#### Promoter motif analysis

The gene IDs were converted into TGACv1 by means of ID History Converter in Ensembl Plants (http://plants.ensembl.org/Triticum_aestivum/Tools/AssemblyConverter) and promoter sequences were collected from Ensembl Plants Biomart (http://plants.ensembl.org/biomart/martview). An in-house developed *cis-*acting element database was used to map annotated promoter motifs. CLC genomics workbench 9.5.3 was used as the analysis tool for searching and alignment.

### Statistical analysis of agronomic traits and protein parameters data

The data for agronomic traits and protein parameters were analysed by R. One-way analysis of variance (ANOVA) was used to determine the significance of sulphur treatments on agronomic traits and protein parameters. Significant statistical differences were judged at a *P* ≤ 0.05.

## Electronic supplementary material

Supplementary figure and table

supplementary table S2

supplementary table S3
