# Supplementary material for: Impact of mid-season sulphur deficiency on wheat nitrogen metabolism and biosynthesis of grain protein
Source: Sci Rep. 2018 Feb 6;8:2499. doi: 10.1038/s41598-018-20935-8 (PMC5802717; doi:10.1038/s41598-018-20935-8)
Supplement: Supplementary file 1 — Supplementary figure and table [file 41598_2018_20935_MOESM1_ESM.pdf]

*MS for Scientific Reports*

# Impact of mid-season sulphur deficiency on wheat nitrogen metabolism and biosynthesis of grain protein

Zitong Yu[1], Angela Juhasz[1], Shahidul Islam[1], Dean Diepeveen[1, 2], Jingjuan Zhang[1], Penghao Wang[1], Wujun Ma[1, 3*]

[1]State Agricultural Biotechnology Centre, School of Veterinary and Life Science, Murdoch University, Perth, WA 6150, Australia.

[2]Western Australian Department of Agriculture & Food, 3 Baron-Hay Ct, South Perth WA 6151, Australia.

[3]Australia-China Joint Centre for Wheat Improvement, Murdoch University, Perth, WA 6150, Australia.

Contact information:

Zitong Yu: Z.Yu@murdoch.edu.au

Angela Juhasz: juhaszangi@gmail.com

Shahidul Islam: S.Islam@murdoch.edu.au

Dean Diepeveen: dean.diepeveen@agric.wa.gov.au

Jingjuan Zhang: J.Zhang@murdoch.edu.au

Penghao Wang: P.Wang@murdoch.edu.au

Wujun Ma: W.Ma@murdoch.edu.au

[*]Corresponding author: Wujun Ma.

## Supplementary Figure S1

Sequence alignment of *GS* and nitrogen metabolism highly enriched DEGs. (a) *GS1*. (b) *GS2*.

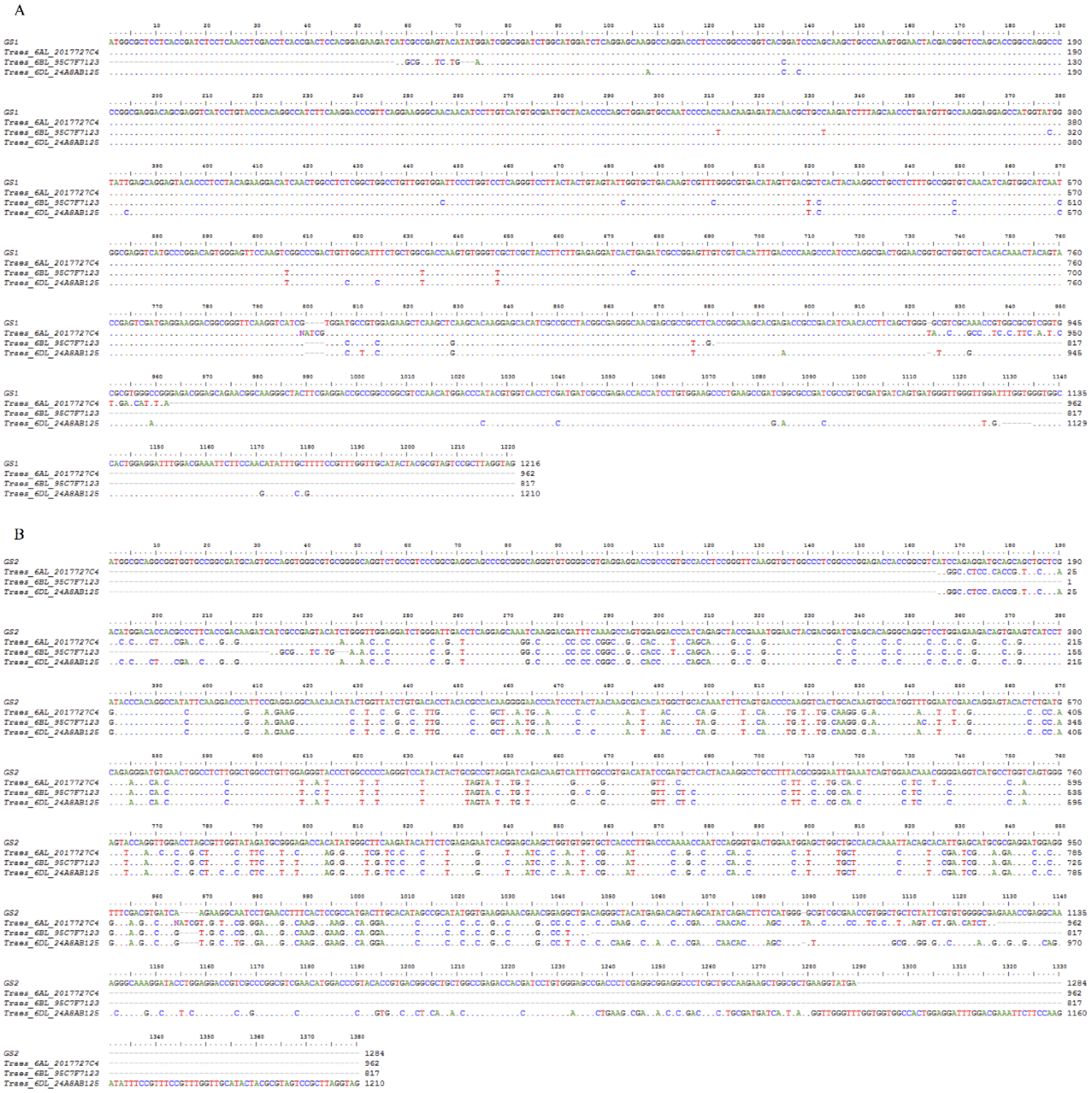

**Supplementary Table S1**

Table S1. The identification of 63 DEGs from the comparison between S30 and S0 at 7 days post-anthesis

| HS vs LS | Gene_ID | Swiss-Prot | Gene_ID | Swiss-Prot |
|---|---|---|---|---|
| | Traes_1AS_6E5DECDA1 | na | Traes_4AL_205E88570 | na |
| | Traes_1AS_E83292840 | na | Traes_4AL_A10FB6994 | Bisdemethoxycurcumin synthase, OS=*O. sativa* |
| | Traes_1BS_68F77FAE7 | patatin | Traes_4BS_64FB912EE | xyloglucan endotransglucosylase/hydrolase |
| | Traes_1BL_D6E1CD173 | non-specific lipid-transfer protein | Traes_4BL_34FE2C7F2 | Protein DROOPING LEAF, OS=*O. sativa* |
| | Traes_1DL_B466DF47A | protein ECERIFERUM 1, OS=*A. thaliana* | Traes_4DL_5612CF456 | Glyceraldehyde-3-phosphate dehydrogenase B, chloroplastic, OS=*P. sativum* |
| | Traes_1DS_812AFD33F | patatin-like protein 1, OS=*O. sativa* | Traes_4DL_5C0CDD31F | apyrase 3, OS=*O. sativa* |
| Down-regulation | Traes_1DS_8D70617FC | patatin | Traes_4DL_B1B379CB5 | Aspartic proteinase Asp1, OS=*O. sativa* |
| | Traes_1DS_FA495B931 | na | Traes_5AL_B446A07423 | non-specific lipid-transfer protein 3 |
| | Traes_2AL_6DEEE3C3E | beta-fructofuranosidase, insoluble isoenzyme 2, OS=*O. sativa* | Traes_5BS_BA0EAE97A1 | na |
| | Traes_2AL_CB3FF487A | ribosomal protein S17, chloroplastic, OS=*Z. mays* | Traes_5BL_304FAFA26 | Lipoxygenase |
| | Traes_2BS_6762A1AAE | taxadien-5-alpha-ol O-acetyltransferase, OS=*T. cuspidata* | Traes_5BL_ED521656E1 | 3-ketoacyl-CoA synthase |
| | Traes_2BS_B8E7189CF | basic endochitinase A, OS=*S. cereale* | Traes_5DS_94D21BF47 | na |
| | Traes_2BS_B8E7189CF1 | basic endochitinase A, | Traes_5DL_488FBAEEC | non-specific lipid-transfer |

| | | | |
|---|---|---|---|
| | OS=*S. cereale* | | protein 3 |
| Traes_2BL_032A12DB3 | ultraviolet-B receptor UVR8, OS=*A. thaliana* | Traes_5DL_7575B5B36 | Dirigent protein 1, OS=*A. thaliana* |
| Traes_2BL_4AAB8719F | ultraviolet-B receptor UVR8, OS=*A. thaliana* | Traes_5DL_8F68FBC81 | non-specific lipid-transfer protein |
| Traes_2BL_7911433A3 | alpha-glucosidase, OS=*O. sativa* | Traes_6AS_33690B236 | Glucomannan 4-beta-mannosyltransferase 1, OS=*O. sativa* |
| Traes_2BL_928000A2B | ultraviolet-B receptor UVR8, OS=*A. thaliana* | Traes_6AL_2017727C4 | Glutamine synthetase |
| Traes_2DL_04892661A | Aldehyde dehydrogenase family 3 member F1, OS=*A. thaliana* | Traes_6AL_38E8DCF06 | Peroxidase |
| Traes_2DL_1B3AD9F4E | Beta-fructofuranosidase, insoluble isoenzyme 2, OS=*O. sativa* | Traes_6BL_34AF8C436 | Peroxidase |
| Traes_2DL_3C229EB92 | protochlorophyllide reductase A, chloroplastic | Traes_6BL_95C7F7123 | Glutamine synthetase |
| Traes_2DL_80DD45E69 | glyceraldehyde-3-phosphate dehydrogenase A, chloroplastic, OS=*Z. mays* | Traes_6DS_3522B8EF6 | Catalase |
| Traes_2DL_85AFEAF70 | non-specific lipid-transfer protein | Traes_6DS_C0358414B | ABC transporter G family member 11, OS=*A. thaliana* |
| TRAES3BF004400040CFD | non-specific lipid-transfer protein | Traes_6DL_0A0ABD677 | Protein TSS, OS=*A. thaliana* |
| TRAES3BF005200020CFD | domain-containing protein | Traes_6DL_24A8AB125 | Glutamine synthetase, GS1 |
| TRAES3BF012600130CFD | esterase/lipase, OS=*A. thaliana* | Traes_6DL_3EF3193D0 | Peroxidase |
| TRAES3BF027700190CFD | sigma factor binding protein 1, chloroplastic, OS=*A. thaliana* | Traes_7AL_291471D52 | Tryptophan synthase beta chain 2, OS=*A. aeolicus* |
| TRAES3BF0678 | 2-alkenal | Traes_7AL_9AE | UPF0481 protein |

| | | | | |
|---|---|---|---|---|
| | 00180CFD | reductase (NADP(+)-dependent), OS=N. tabacum | C84938 | At3g47200, OS=*A. thaliana* |
| | TRAES3BF0763 00030CFD | na | Traes_7BS_5FE2 61AAA | UDP-glucosyltransferase BX8, OS=*Z. mays* |
| | TRAES3BF0883 00010CFD | non-specific lipid-transfer protein | Traes_7DS_71A DD7C7C | na |
| | TRAES3BF0883 00030CFD | non-specific lipid-transfer protein | Traes_7DS_A934 D9C24 | Bisdemethoxycurcumin synthase, OS=*O. sativa* |
| | TRAES3BF0884 00020CFD | non-specific lipid-transfer protein | | |
| | Traes_3DS_E756 029E7 | glutamate decarboxylase | | |
| Up-regulation | Traes_5BS_B432 6E4BD | Pyrophosphate-fructose 6-phosphate 1-phosphotransferase subunit alpha, OS=*R. communis* | | |

**Supplementary Table S4**

Table S4. Identification of *cis*-acting element conserved regions in promoter sequence of three DEGs annotated as glutamine synthetase

| | Gene ID | Name | Region | Match | Accuracy (%) |
|---|---|---|---|---|---|
| AP2/EREBP | Traes_6AL_2017727C4 | AP2/EREBP | 741..744 | ACGT | 100 |
| | Traes_6AL_2017727C4 | AP2/EREBP | 804..807 | ACGT | 100 |
| | Traes_6AL_2017727C4 | AP2/EREBP | 901..906 | GCCGCC | 100 |
| | Traes_6AL_2017727C4 | AP2/EREBP | complement(741..744) | ACGT | 100 |
| | Traes_6AL_2017727C4 | AP2/EREBP | complement(804..807) | ACGT | 100 |
| | Traes_6BL_95C7F7123 | AP2/EREBP | 744..747 | ACGT | 100 |
| | Traes_6BL_95C7F7123 | AP2/EREBP | 812..815 | ACGT | 100 |
| | Traes_6BL_95C7F7123 | AP2/EREBP | 905..910 | GCCGCC | 100 |
| | Traes_6BL_95C7F7123 | AP2/EREBP | complement(744..747) | ACGT | 100 |
| | Traes_6BL_95C7F7123 | AP2/EREBP | complement(812..815) | ACGT | 100 |
| | Traes_6DL_24A8AB125 | AP2/EREBP | 726..729 | ACGT | 100 |
| | Traes_6DL_24A8AB125 | AP2/EREBP | 794..797 | ACGT | 100 |
| | Traes_6DL_24A8AB125 | AP2/EREBP | 894..899 | GCCGCC | 100 |
| | Traes_6DL_24A8AB125 | AP2/EREBP | complement(726..729) | ACGT | 100 |
| | Traes_6DL_24A8AB125 | AP2/EREBP | complement(794..797) | ACGT | 100 |
| ARF | Traes_6AL_2017727C4 | ARF | 738..742 | GAGAC | 100 |
| | Traes_6BL_95C7F7123 | ARF | 741..745 | GAGAC | 100 |
| | Traes_6DL_24A8AB125 | ARF | 723..727 | GAGAC | 100 |
| bZIP | Traes_6AL_2017727C4 | bZIP | 740..745 | GACGTC | 100 |
| | Traes_6AL_2017727C4 | bZIP | 741..744 | ACGT | 100 |
| | Traes_6AL_2017727C4 | bZIP | 804..807 | ACGT | 100 |
| | Traes_6AL_2017727C4 | bZIP | complement(740..745) | GACGTC | 100 |

| | | | | | |
|---|---|---|---|---|---|
| | Traes_6AL_2017727C4 | bZIP | complement(741..744) | ACGT | 100 |
| | Traes_6AL_2017727C4 | bZIP | complement(804..807) | ACGT | 100 |
| | Traes_6BL_95C7F7123 | bZIP | 743..748 | GACGTC | 100 |
| | Traes_6BL_95C7F7123 | bZIP | 744..747 | ACGT | 100 |
| | Traes_6BL_95C7F7123 | bZIP | 812..815 | ACGT | 100 |
| | Traes_6BL_95C7F7123 | bZIP | complement(743..748) | GACGTC | 100 |
| | Traes_6BL_95C7F7123 | bZIP | complement(744..747) | ACGT | 100 |
| | Traes_6BL_95C7F7123 | bZIP | complement(812..815) | ACGT | 100 |
| | Traes_6DL_24A8AB125 | bZIP | 725..730 | GACGTC | 100 |
| | Traes_6DL_24A8AB125 | bZIP | 726..729 | ACGT | 100 |
| | Traes_6DL_24A8AB125 | bZIP | 794..797 | ACGT | 100 |
| | Traes_6DL_24A8AB125 | bZIP | complement(725..730) | GACGTC | 100 |
| | Traes_6DL_24A8AB125 | bZIP | complement(726..729) | ACGT | 100 |
| | Traes_6DL_24A8AB125 | bZIP | complement(794..797) | ACGT | 100 |
| | Traes_6AL_2017727C4 | SURE | 738..742 | GAGAC | 100 |
| SURE | Traes_6BL_95C7F7123 | SURE | 741..745 | GAGAC | 100 |
| | Traes_6DL_24A8AB125 | SURE | 723..727 | GAGAC | 100 |

**Supplementary Table S5**

Table S5. Real time PCR primer for *GS1* and *GS2*

| Genes | Orientation | 5′-3′ sequence |
|---|---|---|
| *GS1* | Forward | GTGGATGCCGTGGAGAAG |
| | Reverse | GCTGAAGGTGTTGATGTCG |
| *GS2* | Forward | CTCGTCCGCGTCCTTGTCCG |
| | Reverse | GCCGACCTGCCCCGCACG |
